# A systematic review of dermatologic manifestations among adult patients with COVID‐19 diagnosis

**DOI:** 10.1002/ski2.20

**Published:** 2021-03-19

**Authors:** L.N. Schwartzberg, S. Advani, D.C. Clancy, A. Lin, J.L. Jorizzo

**Affiliations:** ^1^ New York Institute of Technology College of Osteopathic Medicine New York USA; ^2^ Terasaki Institute of Biomedical Innovation Los Angeles California USA; ^3^ Department of Dermatology St. John's Episcopal Hospital New York USA; ^4^ Department of Dermatology Wake Forest Baptist Health Winston‐Salem North Carolina USA; ^5^ Weill Cornell Medicine Dermatology New York USA

**Keywords:** evidence‐based dermatology, infection, virology

## Abstract

**Background:**

Infection with COVID‐19 is characterized by respiratory, gastrointestinal and neurologic symptoms. However, limited evidence exists of the involvement of the integumentary system among COVID‐19 patients and evidence suggests that these symptoms may even be the first presenting sign.

**Objective:**

To systematically evaluate the literature published on dermatologic signs of COVID‐19 in order to educate doctors about the dermatologic signs of COVID‐19 infection.

**Methods:**

Lit COVID, World Health Organization COVID‐19 database and PubMed were searched using terminology to identify adult patients with confirmed COVID‐19 infection and dermatologic manifestations of disease. The last search was completed on 13 July 2020.

**Results:**

There were 802 reports found. After exclusion, 20 articles were found with 347 patients with confirmed COVID‐19 infection. Within these articles, 27 different skin signs were reported.

**Limitations:**

Limitations of this review include the recency of COVID‐19 infection; so, there are limited published reports and that many reports are not by dermatologists, and so, the cutaneous signs may be misdiagnosed or misdescribed.

**Conclusion:**

Dermatologic manifestations of COVID‐19 may be the first presenting sign of infection; so, dermatologists and doctors examining the skin should be aware of the virus's influence on the integumentary system in order to promptly diagnose and treat the infected patients.

## Graphical abstract

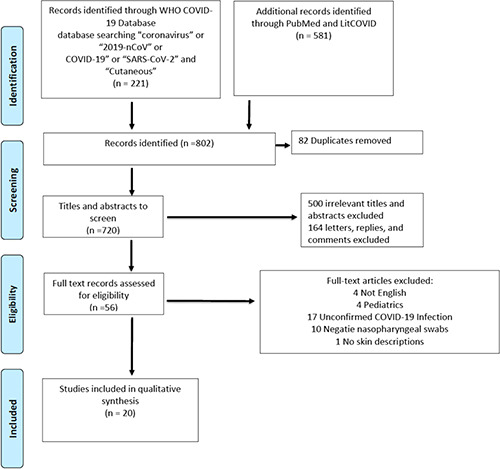

COVID‐19 infection affects multiple organ systems, including the integumentary system. This systematic review explores the reported dermatologic manifestations associated with this infection in adults.

1

**What is already known about this topic?**

- COVID‐19 is a global pandemic with multisystem involvement.

**What does this study add?**

- Identifying COVID‐19 skin signs can facilitate early diagnosis.

## 2 INTRODUCTION

The World Health Organization (WHO) declared COVID‐19 a global health emergency and a pandemic that has led to over 104 million cases and 2.2 million deaths as of 4 February 2021.^1^ Typical symptoms include cough, fever, sore throat and abdominal pain, but dermatologic signs can also occur. These dermatologic manifestations could play a key role in early identification and treatment of COVID‐19. Few case reports of patients with COVID‐19 have highlighted the presence of atypical manifestations of COVID‐19 including dermatologic manifestations.^2^ Since COVID‐19 is still new, there is not a full range of categories of evidence‐based reports on the dermatologic publications thus far.

Commonly published dermatologic manifestations of COVID‐19 include morbilliform, vesiculopapular, pernio‐like lesions and purpura.^3^ The possible underlying pathology is unclear, but emergency data show that the virus leads to release of large quantities of pro‐inflammatory cytokines leading to many downstream effects.^4^

However, given the varied presentation of COVID‐19 patients globally, there exists a need to critically evaluate the dermatologic manifestations associated with COVID‐19 in the rapidly evolving literature. To address this gap, we performed a systematic review of the literature to summarize cutaneous manifestations associated with COVID‐19.

## 3 METHODS

We performed a comprehensive systematic review of clinical and pathologic characteristics of cutaneous signs of COVID‐19 in adults. The systematic review was registered with PROSPERO (registration number CRD42020195935). The included studies had to describe patients who were diagnosed with COVID‐19 and had dermatologic symptoms in patients over 18 years old. We excluded studies that suspected SARS‐CoV‐2 without laboratory confirmation or did not specify whether COVID‐19 infection was confirmed or not. Additionally, studies that did not have a clear description of the cutaneous signs or symptoms in their clinical presentations were excluded. We further excluded review articles, conference proceedings, commentaries and expert opinions were excluded. Lastly, we limited articles to those published in English, in peer‐reviewed journals. We used the Preferred Reporting Items for Systematic Reviews and Meta‐Analyses (PRISMA) guidelines for conducting our review.^5^

### 3.1 Search strategy

The PubMed (National Library of Medicine), LitCOVID (PubMed's COVID‐19 database) and WHO COVID‐19 databases were searched for relevant literature. The last date of the search was 13 July 2020. The main concepts searched were the clinical and pathologic dermatologic characteristics of COVID‐19‐infected patients. A combination of medical subject heading (MeSH) terms and terms included in the title, abstract and keywords were used to develop the search. Search terms included ‘dermatologic’ or ‘skin’ or ‘cutaneous’ combined with ‘COVID‐19’ or ‘SARS‐CoV‐2’ or ‘coronavirus’. This search was then adapted for each individual database.

### 3.2 Study selection

Two authors (LS, DC) independently screened all titles and abstracts, blinded to authors and journal titles, using an Excel workbook.^6^ Data were compiled in a single Excel workbook and consensus was reached on items in which there was disagreement. Articles considered for inclusion were independently reviewed by the two authors and consensus reached by discussion on any disagreements for inclusion. The Excel workbooks also served as our primary tool for gathering all search strategy data and for the creation of the PRISMA flowchart.^5^

### 3.3 Data abstraction

The primary author (LS) identified key study characteristics, including study title, first author, number of confirmed COVID‐19 patients and country. The author then extracted information on the number of patients with skin lesions, description of the cutaneous signs, histopathology results, timeline of symptoms, pertinent medications and prognosis.

### 3.4 Bias and quality assessment

One author (SA) independently rated the quality of included studies using the National Institutes of Health Quality Assessment Tool for Case Series Studies.^7^ This tool assesses the quality of studies on nine key questions including (a) Was the study question or objective clearly stated?; (b) Study population clearly and fully described including a case definition; (c) Were cases consecutive?; (d) Were subjects comparable?; (e) Was the intervention clearly described; (f) Was the outcome measured clearly defined, valid, reliable and implemented consistently?; (g) Was length of follow up adequate; (h) Were the statistical methods well described?; (i) Were results well described? The review rated each of the included article and reported their responses as Yes, No, CD = Cannot Determine, NR = Not Reported or NA = Not Applicable. Further based on their assessment, the reviewers are also asked to rate the overall rating of the article as good, fair or poor.

## 4 RESULTS

### 4.1 Screening process

**FIGURE 1:**
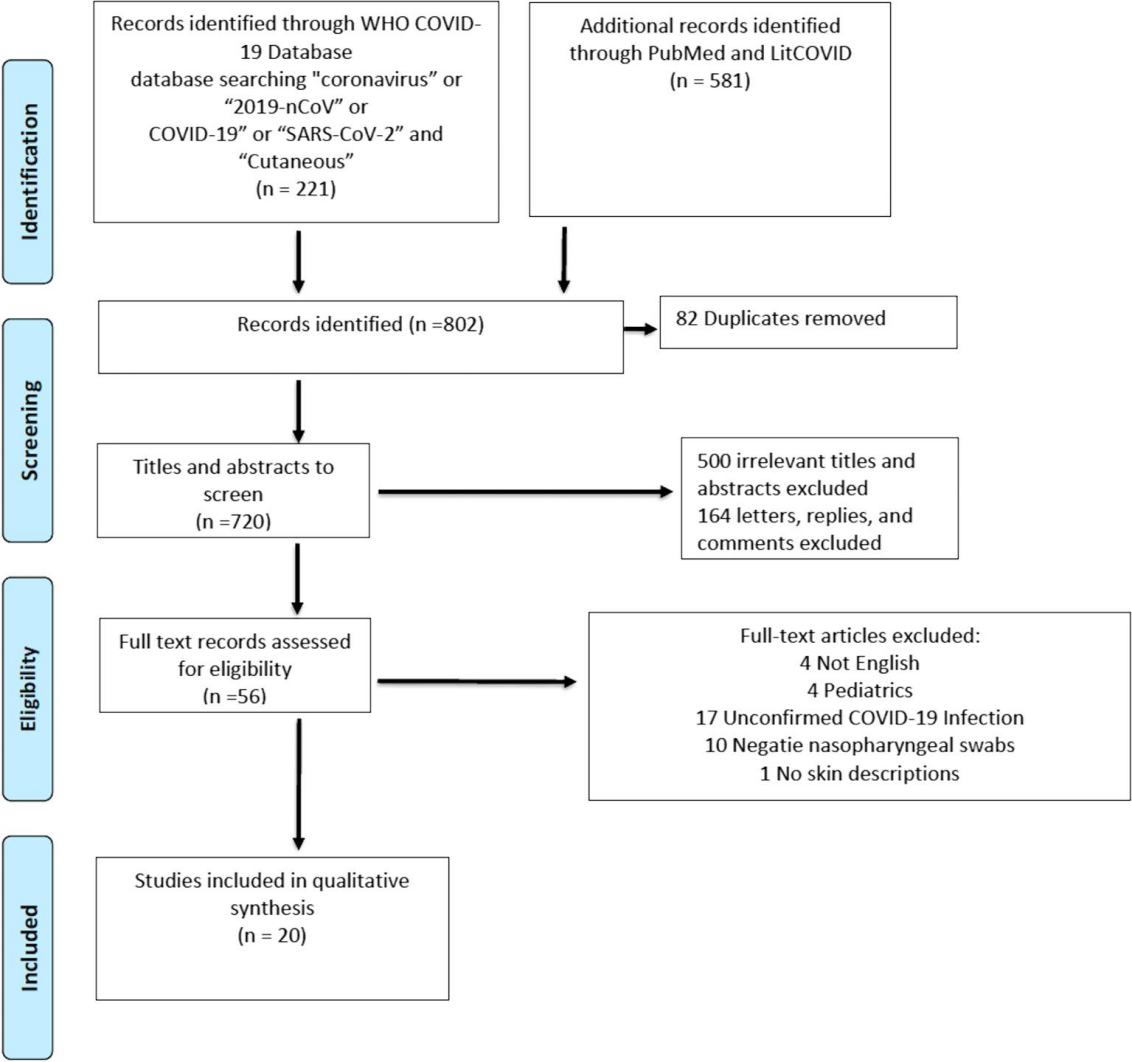
PRISMA Flow Diagram

Our search identified 802 records. After the removal of duplicates, two screeners (LS, DC) screened 720 titles and abstracts and identified 220 articles for full‐text review. Figure 1 provides a PRISMA flowchart of the screening process. Of total 220 screened, 17 articles did not report confirmed COVID‐19 infection, 10 articles had confirmed negative nasopharyngeal swabs, 164 articles were excluded because they were letters, comments or replies (i.e., not original articles), 4 articles were excluded because they were not in English, 1 article did not describe the skin lesions and 4 articles described solely paediatric patients. The final sample consisted of 20 publications for overall analysis.^8^, ^9^, ^10^, ^11^, ^12^, ^13^, ^14^, ^15^, ^16^, ^17^, ^18^, ^19^, ^20^, ^21^, ^22^, ^23^, ^24^, ^25^, ^26^, ^27^

### 4.2 Patient population

The 20 included articles represented a total of 11 case reports,^8^, ^9^, ^10^, ^13^, ^14^, ^15^, ^16^, ^23^, ^24^, ^25^, ^27^ 7 case series,^11^, ^12^, ^17^, ^18^, ^19^, ^20^, ^21^ one observational study^22^ and one prospective study.^26^ Our study only includes the patients reported from these studies who had confirmed COVID‐19 infection, which lead to 347 total patients. Some of these studies reported patients who had confirmed infection, but did not have dermatologic signs.^11^, ^19^, ^22^ After excluding patients who did not have both confirmed infection and at least one dermatologic manifestation, our overall sample size was 255. The mean age reported was 56.92 years old. There were 137 (56.61%) females and 105 (43.80%) males. This excludes one study of 13 patients that did not disclose the gender of those with dermatologic signs. This data can be found in Table 1.

**TABLE 1:** Study information and patient population

First author	Country	Type of article	Number of patients^a^	How patients tested positive	Age	Gender
Hassan, K	Scotland	Case Report	1	RT‐PCR	46	F
Suter, P	Switzerland	Case Report	1	RT‐PCR	42	M
Shanshal, M	Iraq	Case Report	1	RT‐PCR	35	F
Lorenzo‐Villalba, N	France	Case Series	2	RT‐PCR	57	F
Freeman, E	International Registry	Case Series	23	(14) PCR, (5) antibody testing, and (4) unknown assay	41 median	11 F12 M
Lagziel, T	USA	Case Report	1	RT‐PCR	58	F
Eka Putraa, B	Indonesia	Case Report	1	RT‐PCR	29	M
Sakaida, T	Japan	Case Report	1	RT‐PCR	52	F
de‐Medeiros, V	Brazil	Case Report	1	RT‐PCR	55	F
Rivera‐Oyola, R	USA	Case Series	2	SARS‐CoV‐2 rapid respiratory panel	6060	MF
Gianotti R	Italy	Case Series	3	RT‐PCR	598989	FFM
Magro C	USA	Case Series	3	RT‐PCR	326640	M*F*F
Sachdeva M	Italy	Case Series	3	RT‐PCR	717772	*F* *F*F
Freeman, E	International Registry	Case Series	171	RT‐PCR	44 median	93 F78 M
Dalal, A	India	Observational Study	13	RT‐PCR	39.3 mean	Unspecified
Elsaie, ML	Saudi Arabia	Case Report	1	RT‐PCR	57	M
Ho, B	United Kingdom	Case Report	1	RT‐PCR	79	F
Papamichalis, P	Greece	Case Report	1	RT‐PCR	68	M
Fernandez‐Nieto, L	Spain	Prospective Study	24	RT‐PCR	49.5 median	18 F6 M
Diaz‐Guimaraens, B	Spain	Case Report	1	RT‐PCR	48	M

^a^ Only includes patients reported in the study who were confirmed COVID‐19 positive and had dermatologic signs.

### 4.3 Cutaneous signs

**FIGURE 2:**
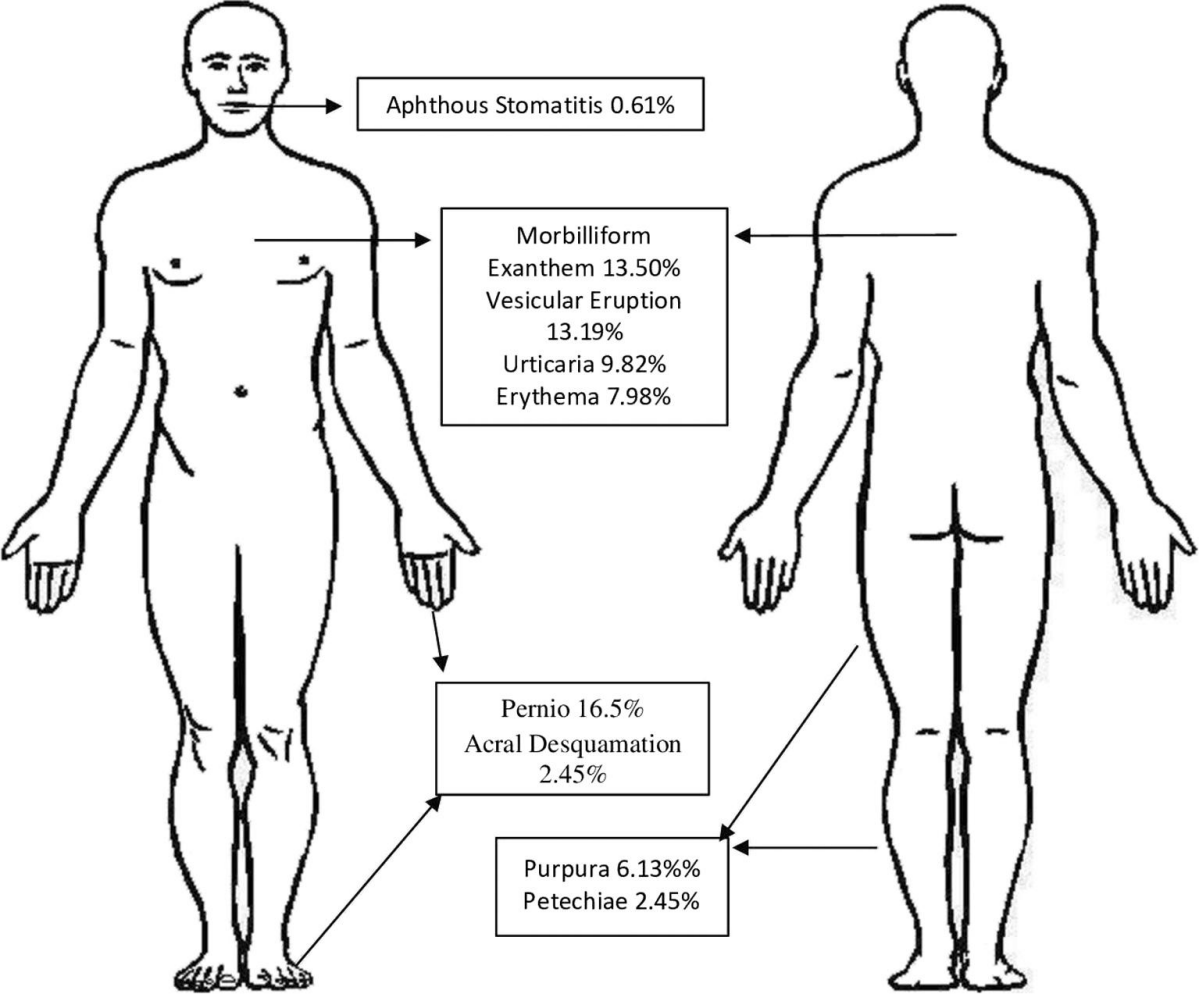
Frequency of Cutaneous Findings Illustrated in Their Common Anatomic Locations

Common skin manifestations reported in confirmed COVID‐19 patients include pernio‐like lesions, morbilliform exanthems and vesicular cutaneous eruptions. The number of reported cases of each skin sign can be seen in Table 2. Pernio‐like lesions affect the acral areas such as fingers and toes. They are a violaceous discoloration of the skin that can be associated with pruritis, burning or blistering. Morbilliform exanthems are a type of viral exanthem that results in rose‐coloured macules and papules that may coalesce. Vesicular cutaneous eruptions resemble a chickenpox‐like exanthem that may present with monomorphic vesicles or vesicles at various stages. Figure 2 demonstrates many of these findings that have been reported.

**TABLE 2:** Number of patients with each cutaneous sign

Dermatologic diagnosis	Number of patients with skin sign	Percentage of patients with skin sign
Pernios	54	16.56%
Morbilliform exanthem	44	13.50%
Vesicular eruption	43	13.19%
Urticaria	32	9.82%
Erythematous exanthem	26	7.98%
Papulosquamous eruption	18	5.52%
Retiform purpura	12	3.68%
Livedo reticularis‐like lesions	9	2.76%
Grover's‐like eruption	9	2.76%
Symptom of pruritis without physical sign	8	2.45%
Acrocyanosis	8	2.45%
Purpura	8	2.45%
Acral desquamation	8	2.45%
Petechiae	8	2.45%
Dengue‐like eruption	8	2.45%
Pressure ulcers	6	1.84%
Livedo racemosa	5	1.53%
Bullous dermatitis	4	1.23%
Erythroderma	4	1.23%
Erythema nodosum	3	0.92%
Aphthous stomatitis	2	0.61%
Milaria rubra	2	0.61%
Telogen effluvium	1	0.31%
Transient acantholytic dermatosis	1	0.31%
Herpes zoster	1	0.31%
Multisystem inflammatory syndrome	1	0.31%
Acneiform eruption	1	0.31%

### 4.4 Location of skin signs

**FIGURE 3:**
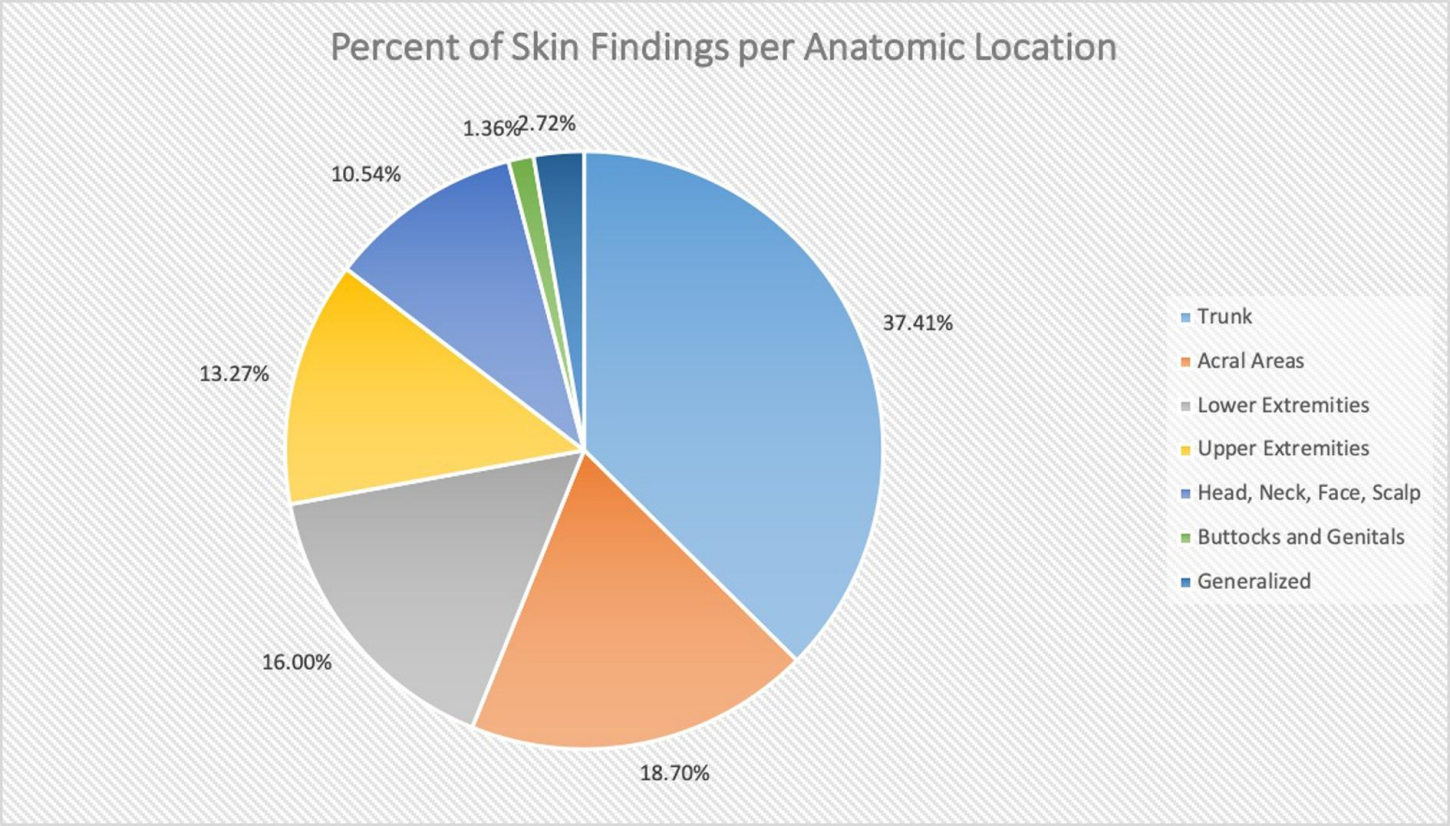
Percent of Skin Findings per Anatomic Location

Many patients had more than one body site affected by the cutaneous manifestations of COVID‐19. The most common location for a dermatologic sign was the trunk (including abdomen, back and chest); 37.41% of dermatologic findings were reported on the trunk. Following that, the second most common site was the lower extremities; 16.00% of cutaneous signs were found on the legs. Cumulatively, the acral areas, including palms, soles, hands, feet, fingers and toes, made up a total of 18.70% of skin findings. A generalized distribution was seen in 2.72% of skin findings. The head and neck involved 5.78% of findings and the genitals, buccal mucosa and buttocks comprised 2.38% of cutaneous signs. Figure 3 demonstrates where each cutaneous sign was found.

### 4.5 Timeline of skin signs

The most common timeline of symptoms was dermatologic signs appearing two weeks after COVID‐19 symptom onset. The onset of skin signs in relation to other COVID‐19 symptoms varied across studies. Skin signs preceded other viral symptoms in eight patients. Twelve patients presented with skin signs at the time of other COVID‐19 symptoms. Skin signs were the only manifestation of infection in seven patients, meaning these patients were otherwise asymptomatic for the entirety of the viral infection. There were six studies that reported a nonspecific timeline of dermatologic signs in relation to COVID‐19 symptoms.

Similar to symptom timelines, the duration of dermatologic signs varied greatly. Two cases reported resolution within 1 day. Of the 16 studies that reported a resolution of skin signs, the mean duration was 6.06 days.

### 4.6 Course of disease

Three patients died from viral infection prior to dermatologic disease resolution. Two of which had pernio‐like lesions and one with urticaria.^12^, ^24^ Another study reported pernio‐like lesions to only be in patients with mild disease and retiform purpura in more severe, hospitalized patients.^21^ One patient with acral ischemia died 40 days after viral resolution due to sepsis and co‐morbid AML.^25^ Another patient with palmoplantar purpura entered a comatose state and experienced a stroke.^19^ A patient with petechiae, an erythematous exanthem and aphthous ulcers was last reported as being transferred to the ICU at another hospital.^15^ Six articles did not discuss prognosis or final outcome. The rest of the patients were last reported as recovering or reached complete recovery.

### 4.7 Dermatopathology

Out of the 20 studies, nine had biopsy specimen to report.^12^, ^13^, ^15^, ^17^, ^18^, ^19^, ^26^, ^27^ Of those 9 studies, 28 biopsies were taken and examined. Histopathology results can be seen in Table 3. Some common findings included a vacuolar interface dermatitis, thrombogenic vasculopathy and complement deposition within the dermis. Other non‐specific pattern seen included perivascular lymphocytic infiltration and epidermal necrosis.

**TABLE 3:** Biopsy specimen and dermatopathology

First author	Cutaneous diagnosis	Skin biopsy
Freeman, E	Pernios (1)	Mild vacuolar interface dermatitis with dense superficial and deep lymphocytic inflammation, consistent with pernio versus connective tissue disease. No thrombi were noted.
Lagziel, T	Bullous interface dermatitis (1)	Detached epidermis with a ‘basket‐weave’ stratum corneum, separated at the dermal‐epidermal junction. Spongiosis and basilar vacuolar changes with rare dyskeratotic cells were present. Superficial dermal edema was also present
Sakaida, T	Erythematous cutaneous eruption (1)	slight liquefaction with perivascular and periadnexal mixed cell infiltrations from the papillary dermis to the deep subcutaneous tissue
	Petechiae (1)	Liquefaction and perivascular mixed cell infiltrations, including histiocytes and neutrophils
Rivera‐Oyola, R	Viral exanthem (1)	Mild perivascular infiltrate of mononuclear cells surrounding vessels in the superficial dermis. The epidermis showed scattered foci of hydropic changes, along with minimal acanthosis, slight spongiosis, and foci of parakeratosi
Gianotti R	Viral exanthem (2)	Superficial perivascular dermatitis with slight lymphocytic exocytosis. Swollen thrombosed blood vessels with neutrophils, eosinophils and nuclear debris were found within the dermis
	Transient Acantholytic Dermatosis (1)	Superficial perivasascular vesicular dermatitis with nests of Langerhans cells within the epidermis. Focal acantholytic suprabasal clefts, dyskeratotic and ballooning keratinocytes and a patchy band‐like infiltration with occasional necrotic keratinocytes
Magro C	Retiform purpura (1)	Thrombogenic vasculopathy with extensive necrosis of the epidermis and adnexal structures, including the eccrine coil. Interstitial and perivascular neutrophilia with leukocytoclasia. Considerable depositions of C5b‐9 within microvasculature
	Purpura (1)	Superficial vascular ectasia and an occlusive arterial thrombus within the reticular dermis. No surrounding inflammation. Extensive vascular deposits of C5b‐9, C3d and C4d (Figure 6D) within the dermis, with marked deposition in an occluded artery
	Livedo racemosa (1)	Perivascular lymphocytic infiltrate in the superficial dermis with deeper seated small thrombi within rare venules of the deep dermi. No clear vasculitis. Significant vascular deposits of C5b‐9 and C4d
Freeman, E	Retiform purpura (3); livedo racemosa (2), livedo Reticularis (1)	Thrombotic vasculopathy
	Pressure injury (1)	Thrombotic vasculopathy
	Grover's‐like eruption (1)	Granulomatous dermatitis with increased central mucin deposition
	Papulosquamous eruption (2)	Spongiosis and dermal inflammation
	Palpable purpura (1)	Leukocytoclastic vasculitis
	Pernio‐like lesions (1); morbilliform exanthem (2)	Vacuolar interface dermatitis, subepidermal edema, and superficial and deep lymphocytic inflammation
Fernandez‐Nieto, D	Vesicular cutaneous eruption (2)	Intraepidermal vesicles with mild acantholysis and ballooned keratinocytes; epidermal detachment and confluent keratinocytic necrosis, fibrinoid material with acute inflammation
Diaz‐Guimaraens, B	Petechiae (1)	a superficial perivascular lymphocytic infiltrate with abundant erythrocyte extravasation and focal papillary edema, along with focal parakeratosis and isolated dyskeratotic cells. No features of thrombotic vasculopathy were present

### 4.8 Bias and quality assessment

Table 4 summarizes the overall quality of the included studies. Overall of the 20 included studies, we found 10 to be of fair quality and 10 to be of good quality in terms of their evidence and risk of bias.

**TABLE 4:** Quality assessment using the NIH case series assessment tool

First author	Was the study question or objective clearly stated?	Study population clearly and fully described including a case definition	Cases consecutive	Subjects comparable	Intervention clearly described	Outcome measured clearly defined, valid, reliable and implemented consistently	Was length of follow‐up adequate?	Statistical methods well describe?	Were results well described?	Overall rating	
Dalal	Yes	Yes	Yes	Yes	Yes	Yes	Yes	NA	Yes		Good
de‐Mederios	Yes	NA	NA	NA	Yes	Yes	Yes	NA	Yes		Good
Elsaie	Yes	Yes	CD	CD	Yes	Yes	Yes	NA	Yes		Fair
Freeman	Yes	Yes	Yes	Yes	Yes	Yes	Yes	Yes	Yes		Good
Freeman	Yes	Yes	Yes	Yes	Yes	Yes	Yes	Yes	Yes		Good
Gianotti	Yes	No	CD	CD	Yes	Yes	Yes	NA	Yes		Good
Hassan	Yes	Yes	NA	NA	Yes	Yes	CD	NA	Yes		Fair
Ho	Yes	No	CD	CD	Yes	No	CD	NA	Yes		Fair
Khurana	Yes	No	NA	NA	CD	Yes	CD	NA	Yes		Fair
Lagziel	Yes	Yes	NA	NA	Yes	Yes	Yes	NA	Yes		Good
Lorenzo‐Villaba	Yes	Yes	CD	CD	Yes	Yes	CD	NA	Yes		Fair
Magro	Yes	Yes	CD	CD	Yes	Yes	Yes	NA	Yes		Good
Papamichalis	Yes	Yes	NA	NA	Yes	Yes	Yes	NA	Yes		Good
Putra	Yes	Yes	NA	NA	Yes	Yes	CD	NA	Yes		Fair
Rivera‐Oyola	Yes	No	NA	NA	CD	Yes	CD	NA	Yes		Fair
Sachdeva	Yes	No	CD	CD	Yes	No	CD	NA	Yes		Fair
Sakaida	Yes	No	NA	NA	CD	Yes	CD	NA	Yes		Fair
Sanchez	Yes	No	NA	NA	Yes	Yes	CD	NA	Yes		Good
Shanshal	No	Yes	NA	NA	Yes	No	CD	NA	Yes		Fair
Suter	Yes	Yes	NA	NA	Yes	Yes	Yes	NA	Yes		Good

## 5 DISCUSSION

Reports of dermatologic signs of COVID‐19 are on the rise, but most cases have not had laboratory confirmation of SARS‐CoV‐2 infection. Our review includes only laboratory‐confirmed infection in an attempt to avoid coincidental skin findings or medication side effects. Reports of overt medication induced dermatologic conditions in COVID‐19 patients have been excluded, but some reports have noted medications taken near the time of dermatologic signs. Patients on medication create an added difficulty in understanding the pathogenesis behind the skin manifestations of COVID‐19 because of the potential of dermatologic side effects of medications.

The importance of this systematic review is that skin signs may be the only or the first presenting sign of COVID‐19 infection. Diagnosing this atypical presentation of viral infection early helps limit the potential of rampant spread of infection. A limitation of this paper is that COVID‐19 is still relatively new, but future studies can explore if these dermatologic signs are predictive of future complications. Atypical COVID‐19 presentations such as solely cutaneous signs are likely due to a similar pathology as the respiratory symptoms of COVID‐19. The histology of the skin biopsy specimen revealed similar findings to the histopathology found in the lungs of deceased COVID‐19 patients. These overlapping findings included thrombi within the small vessels.^27^ The exact mechanism of vasculopathy that occurs after the virus binds to endothelial cells are not well understood. The existing research postulates that increase release of cytokines occurs after this binding, which leads to haemostatic effects.^28^ Laboratory findings in patients with COVID‐19 that support this pathogenesis, but not consistently. Common laboratory findings include d‐dimers and an occasional decrease in platelet numbers. Contradicting laboratory data has been published and requires further investigation.

Vasculitis occurs when there is a robust inflammation resulting from immune‐mediated complexes that deposit around blood vessels causing destruction, deposition of fibrin and thrombus formation. The dermatologic presentation of vasculitis usually occurs about 1 week after the inciting trigger. In contrast, vasculopathy is caused by a hypercoagulable state that leads to thrombosis and necrosis. Many of the published dermatologic manifestations of COVID‐19 consist of overlap of the two. More recent reports have discussed an urticarial vasculitis associated with the virus.^29^ These findings have important implications to the pathogenesis as well as prognosis of COVID‐19. In our review, most patients with cutaneous signs recovered completely. The patients who did not experience complete resolution had cutaneous manifestations including pernio, erythema and petechiae, retiform purpura, and urticaria.^12^, ^15^, ^19^, ^24^ Retiform purpura was found exclusively in hospitalized patients in a study of 171 patients, 11 of whom had retiform purpura.^21^ This cutaneous manifestation of vessel damage may be indicative of poorer patient outcomes, but as of yet it is too early to tell.

Larger studies that included both patients who had laboratory‐confirmed COVID‐19 infection and those who had only suspected, but unconfirmed, infection were not included in our review. Our study criteria only incorporated reports with confirmed COVID‐19 infection in order to maintain high validity. However, a potential limitation of this inclusion criteria may have led to the exclusion of cutaneous signs associated with milder disease state of COVID‐19. Patients with confirmed or suspected infection have demonstrated similar cutaneous manifestations of disease. In a study of 72 patients, combining confirmed and unconfirmed cases, morbilliform exanthem, livedo reticularis, pernio‐like lesions, urticaria and papulosquamous manifestations were all reported similarly to the results of our review.^20^ One study of 682 patients reported 171 confirmed infections and 511 clinically suspected infections. In comparison to those with unconfirmed infection, those with confirmed infections had fewer reports of pernio‐like lesions (18% vs. 77%) and more reports of morbilliform exanthem (22% vs. 4.9%).^21^

Since the date of our literature search, numerous dermatologic reports have been published. Consistent with our findings, newer publications have reported various morphologies of dermatoses related to COVID‐19 infection, even within the same patient. One COVID‐19‐positive patient was reported to have an evolving eruption which started out as a maculopapular exanthem with vesicular dermatitis on the acral surfaces, but weeks later, tense bullae consistent with classic bullous pemphigoid were developed.^30^ The more recent reports documenting laboratory‐confirmed SARS‐CoV‐2 infection can be seen in Table 5.^30^, ^31^, ^32^, ^33^, ^34^, ^35^, ^36^, ^37^, ^38^, ^39^, ^40^, ^41^, ^42^ Although more reports have emerged, there is still limited evidence‐based reports regarding the predictive prognosis and significance of timeline of these dermatologic signs.

**TABLE 5:** Recent reports excluded in the review

First author	Patient age and gender	Cutaneous manifestation and location	Notes
Goon	82 years old (yo) F	Morbilliform exanthem; vesicles on acral surfaces; tense bullae on the trunk and limbs	Patient started out with the morbilliform exanthem and vesicular dermatitis on the acral surfaces, but weeks later developed tense bullae consistent with classic bullous pemphigoid. Patient also had cardiac comorbidities.
Patel	78 yo F	Morbilliform exanthem; vesicles and urticaria on trunk and malar cheeks	Patient had mild symptoms typical of COVID‐19. One week prior to symptoms, patient developed the cutaneous signs. One week after symptom onset, patient's exanthem began to desquamate.
Cepeda‐Valdes	50yo F and 20yo M	Urticarial vasculitis on lower extremities	Patients were otherwise asymptomatic.
Danarti	50 yo M	Follicular eruption on upper extremities, neck, and trunk	Patient was otherwise asymptomatic.
Bosch‐Amate	71 yo F	Cutaneous small vessel vasculitis on lower extremities	Cutaneous signs started 1 week after COVID‐19 symptoms. Patient was hospitalized for COVID‐19 pneumonia.
Tamai	54 yo M, 24 yo M, 81 yo F	Erythematous papules on trunk and extremities	Patients were otherwise asymptomatic.
Adekiigbe	47yo M	Gangrene of the toes	Notably, the patient also had diabetes and developed the purpuric toes 11 days after symptom onset. The gangrene required management with amputation.
Abasaeed	40yo M	Angioedema of the lips and periorbital area; truncal urticaria	The patient also experienced shortness of breath and fever.
Rubin	27 yo F	Chilblain‐like lesions with hemorrhagic bullae on the bilateral toes	The patient also experienced anosmia and ageusia.
Vanaparthy	63 yo F	Raynaud's phenomenon	The patient also had mild COVID‐19 symptoms.
Iancu	41 yo F	Morbilliform exanthem	Disseminated exanthem erupted 15 days after treatment of COVID‐19. Medications used included hydroxychloroquine, azithromycin, and lopinavir/ritonavir.
Rotman	62 yo F	Calciphylaxis and thrombotic retiform purpura of the lower extremities	Notably, the patient also had end‐stage renal disease. The patient passed away weeks after onset.

## 6 CONCLUSION

Skin manifestations vary drastically in presentation, but this systematic review helps categorize these various morphologies. The dermatologist may be the first to see symptoms in a COVID‐19 patient and should be prepared to identify an associated dermatologic manifestation of COVID‐19. Further research should be done to explore the predictive value of each dermatologic sign.

## CONFLICT OF INTERESTS

No conflict of interests have been declared.
